# lncRNA GAS8-AS1 downregulates lncRNA UCA1 to inhibit osteosarcoma cell migration and invasion

**DOI:** 10.1186/s13018-020-1550-x

**Published:** 2020-02-03

**Authors:** Zhuqing Zha, Qingmin Han, Wenjing Liu, Shaochuan Huo

**Affiliations:** 1 Department of Orthopedics, Luoyang Orthopedic Hospital of Henan Province, Orthopedic Hospital of Henan Province, No.100 Yongping Road, Zhengzhou City, Henan Province 450016 People’s Republic of China; 2 Department of Orthopedics, Guangzhou University of Chinese Medicine Third Affiliated Hospital, Guangzhou City, Guangdong Province 510375 People’s Republic of China; 3 Shenzhen Hospital (Futian) of Guangzhou University of Chinese Medicine, No. 6001, North Ring Road, Futian District, Shenzhen City, Guangdong Province 518048 People’s Republic of China

**Keywords:** Osteosarcoma, lncRNA GAS8-AS1, lncRNA UCA1, Survival, Invasion

## Abstract

**Background:**

Osteosarcoma (OS) is the most common type of primary bone tumor that mainly affects adolescents and young adults. The present study explored the role of lncRNA GAS8-AS1 in OS.

**Methods:**

A total of 48 OS patients were selected from the 82 OS patients admitted by Luoyang Orthopedic Hospital of Henan Province between May 2010 and May 2013. Transient cell transfections, Transwell cell migration and invasion assay, RT-qPCR, and patient follow-up were carried out during the research.

**Results:**

The results showed that GAS8-AS1 was downregulated, while UCA1 was upregulate in cancer tissues in comparison to adjacent non-cancer tissues of OS patients. GAS8-AS1 was not affected by clinical stage. Follow-up study showed that downregulated GAS8-AS1 in cancer tissues was closely correlated with poor survival. GAS8-AS1 and UCA1 were inversely correlated in cancer tissues. Overexpression of UCA1 failed to affect the expression of GAS8-AS1, while overexpression of GAS8-AS1 led to downregulated expression of UCA1 in OS cells, while the molecular mediators between these two lncRNAs are unknown. Overexpression of GAS8-AS1 did not affect OS cell proliferation but significantly inhibited cancer cell migration and invasion. Overexpression of UCA1 promoted the migration and invasion of OS cells and attenuated the effects of overexpressing GAS8-AS1.

**Conclusions:**

Therefore, GAS8-AS1 may inhibit OS cell migration and invasion by downregulating oncogenic UCA1.

## Background

Osteosarcoma (OS) is the most common type of primary bone tumor that mainly affects adolescents and young adults [1]. The development of therapeutic approaches, such as surgical resection, radiotherapy, and combinatorial chemotherapy [2] has significantly improved the overall survival of OS patients over the past several decades [2]. However, only 40% of OS patients are diagnosed at early stages, and the prognosis of patients with advanced OS is generally poor [3]. In addition, post-operative local or distant recurrence will occur in 30–50% of patients with initial localized tumor and more than 80% of patients with metastatic tumor [4, 5]. Treatment outcomes are unsatisfactory for OS largely due to the lacking of fully understanding of the pathogenesis [6]. Therefore, in-depth investigation of molecular mechanisms involved in OS is of great importance.

Studies on the molecular pathogenesis of OS have revealed that genetic factors are the key players in the development and progression of OS [7]. Besides oncogenes and tumor suppressors, long non-coding RNAs (lncRNAs, > 200 nt) have also been identified as critical regulators in cancer biology [8]. lncRNAs participate in cancer development and progression by regulating the expression of oncogenes and tumor suppressors [9]. Therefore, regulation of lncRNAs expression may indirectly contribute to cancer prevention and treatment [10]. lncRNA GAS8-AS1 has been characterized as a tumor suppressor in papillary thyroid carcinoma and colorectal cancer [11–13]. Our preliminary deep sequencing data revealed that GAS8-AS1 was downregulated in OS and was inversely correlated with lncRNA UCA1, which has been identified as an oncogenic lncRNA in OS [14]. Our study was carried out to investigate the interactions between GAS8-AS1 and UCA1 in OS.

## Methods

### Patients information

A total of 48 OS patients (30 males and 18 females, with the age of 12 to 34 years old and the mean age of 23.3 ± 4.8 years old) were selected from 82 OS patients admitted by Luoyang Orthopedic Hospital of Henan Province between May 2010 and May 2013. Inclusion criteria are as follows: (1) patients diagnosed by histopathological examinations, (2) patients agreed to participate in follow-up studies, and (3) patients signed informed consent. Exclusion criteria are as follows: (1) patients complicated with other medical conditions, such as other types of malignancies, chronic diseases, other bone diseases and severe infections and (2) patients received treatment before admission. According to the staging criteria proposed by AJCC, there were 13, 13, 12 and 10 cases at stage I, II, III and IV, respectively. This study was approved by the Ethics Committee of Luoyang Orthopedic Hospital of Henan Province.

### Five-year follow-up

All patients were followed up for 5 years or until their deaths. Follow-up was performed by telephone or outpatient visit. The survival of patients was recorded and patients who were lost before the end of follow-up or died of other causes were excluded from this study.

### Tissue collection and cell lines

OS cancer tissues and the adjacent non-cancer tissues were collected from each patient through biopsy before therapies. All specimens were confirmed by 3 experienced pathologists. The human OS cell lines U2OS and MG-63 were used in this study. Cells of both cell lines were purchased from ATCC (USA). Cells were cultured in Eagle’s Minimum Essential Medium (10% FBS) at 37 °C with 5% CO_2_.

### RT-qPCR

RiboZol™ RNA Extraction Reagent (VWR, USA) was used to extract total RNAs from tissues and cells. Reverse transcription was performed using SuperScript IV Reverse Transcriptase (Thermo Fisher Scientific) with the following conditions: 23 °C for 10 min, 52 °C for 20 min, and 80 °C for 10 min. qPCR systems were prepared using SYBR® Green Realtime PCR Master Mix (Toyobo, Japan). 18S rRNA was used as endogenous control. Primer sequences were 5′-CAACGAGCAAACAAGAAGGA-3′ (forward) and 5′-TGAGCCAAACAGACCAGTCA-3′ (reverse) for GAS8-AS1, 5′-TTTATGCTTGAGCCTTG-3′ (forward) and 5′-CTTGCCTGAAATACTTG-3′ (reverse) for UCA1, and 5′-CTACCACATCCAAGGAAGCA--3′ (forward) and 5′-TTTTTCGTCACTACCTCCCCG-3′ (reverse) for human 18S rRNA. PCR conditions were 95 °C for 1 min, followed by 40 cycles of 95 °C for 10s, and 58 °C for 45 s. All PCR reactions were repeated 3 times and data were processed using 2^−ΔΔCT^ method.

### Transient cell transfection

Expression vectors of GAS8-AS1 (NCBI accession: NR_122031.1) and UCA1 (NCBI accession: NR_015379.3) and empty vector were constructed by Sangon (Shanghai, China) using pcDNA3.1 vector as backbone. GAS8-AS1 and UCA1 expression vectors and empty vectors (negative control) were transfected into U2OS and MG-63 cells using Lipofectamine® 2000 Reagent (Thermo Fisher Scientific). Cells without transfections were used as control cells. Subsequent experiments were performed at 24 h after transfections.

### Transwell migration and invasion assay

At 24 h after transfection, U2OS and MG-63 cells were harvested to prepare single-cell suspensions using non-serum Eagle’s Minimum Essential Medium. Cell density was adjusted to 3 × 10^4^ cells/ml. Cell suspensions were transferred to the upper chamber of the Transwell insert (0.1 ml/well), while the lower chamber was filled with Eagle’s Minimum Essential Medium containing 20% FBS. The upper chamber was coated with Matrigel for overnight before invasion assay to mimic the in vivo condition of cancer cell invasion. After cell culture for 2 h, membranes were collected, cleaned, and stained with 0.5% crystal violet (Sigma-Aldrich, USA) for 20 min at room temperature. Cells were observed and counted under an optical microscope.

### Statistical analysis

Experiments were repeated 3 times. Differences between OS and non-cancer tissues were analyzed by paired *t* test. Differences among multiple clinical stages or among different cell transfection groups were analyzed by one-way ANOVA and Tukey *t* test. Linear regression was used for correlation analysis. Patients were divided into high (*n* = 22) and low (*n* = 26) GAS8-AS1 level groups (Youden’s index, cutoff value = 2.09) based on the expression data in OS tissues. Survival curves were plotted and compared using K-M method and log-rank test. Differences were statistically significant when *p* < 0.05.

## Results

### GAS8-AS1 and UCA1 showed opposite expression patterns in OS tissues

**Fig. 1:**
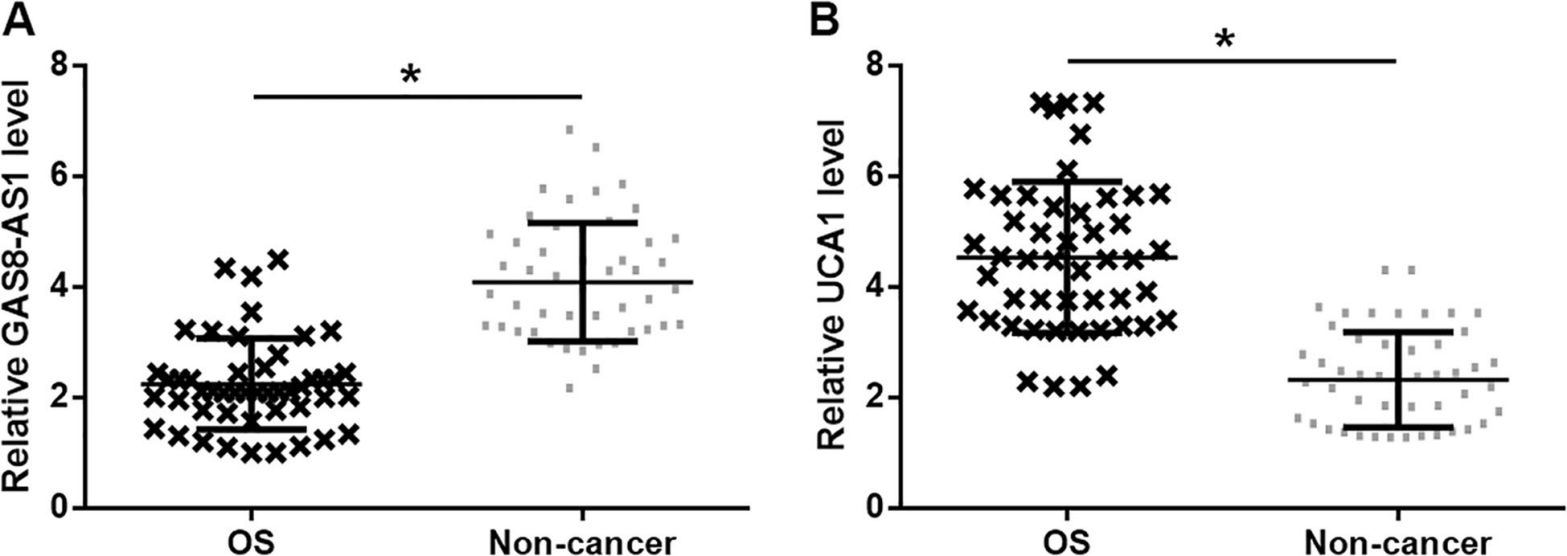
GAS8-AS1 and UCA1 showed opposite expression patterns in OS tissues. Analysis of expression data by paired *t* test showed that GAS8-AS1 was downregulated (**a**), while UCA1 was upregulated (**b**) in cancer tissues in comparison with that in adjacent non-cancer tissues of OS patients (*p < 0.05)

Expression levels of GAS8-AS1 and UCA1 in OS tissues were determined by RT-qPCR, and the expression data were compared by paired *t* test between OS and non-cancer tissues. The results showed that GAS8-AS1 was significantly downregulated (Fig. 1a, *p* < 0.05), while UCA1 was significantly upregulated (Fig. 1b, *p* < 0.05) in OS tissues in comparison to adjacent non-cancer tissues of OS patients.

### Expression of GAS8-AS1 predicted survival

**Fig. 2:**
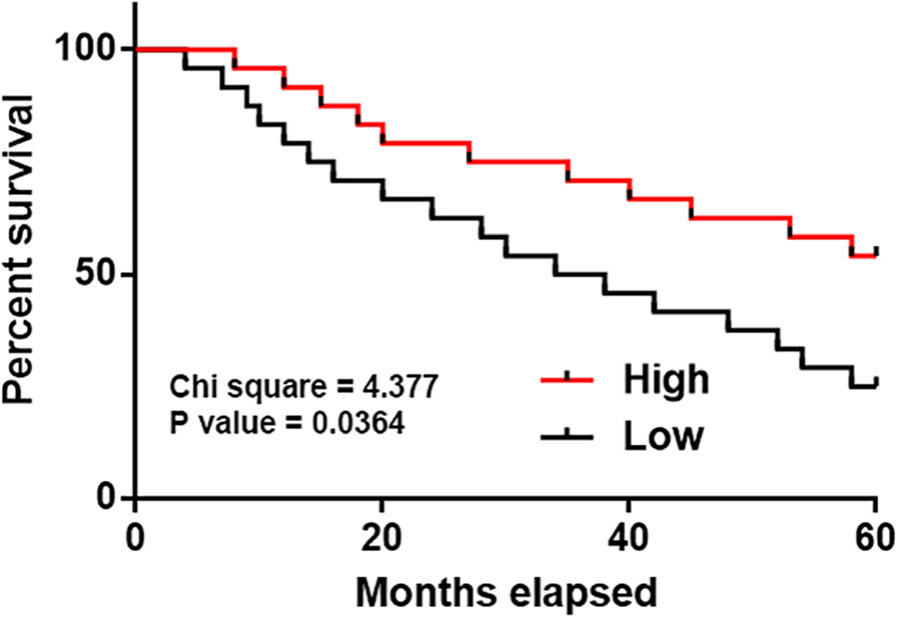
Expression of GAS8-AS1 predicted survival. Survival curve analysis showed that downregulated GAS8-AS1 in OS tissues was closely correlated with poor survival

Expression levels of GAS8-AS1 in OS tissues among OS patients at different clinical stages were compared by one-way ANOVA and Tukey test. It showed that the expression of GAS8-AS1 was not significantly different among patients at different clinical stages (data not shown). Patients were divided into high (*n* = 22) and low (*n* = 26) GAS8-AS1 level groups (Youden’s index). Survival curves were plotted and compared using K-M method and log-rank test. Follow-up study showed that patients with low level of GAS8-AS1 in OS tissues had worse survival conditions (Fig. 2).

### GAS8-AS1 and UCA1 were inversely correlated in OS tissues

**Fig. 3:**
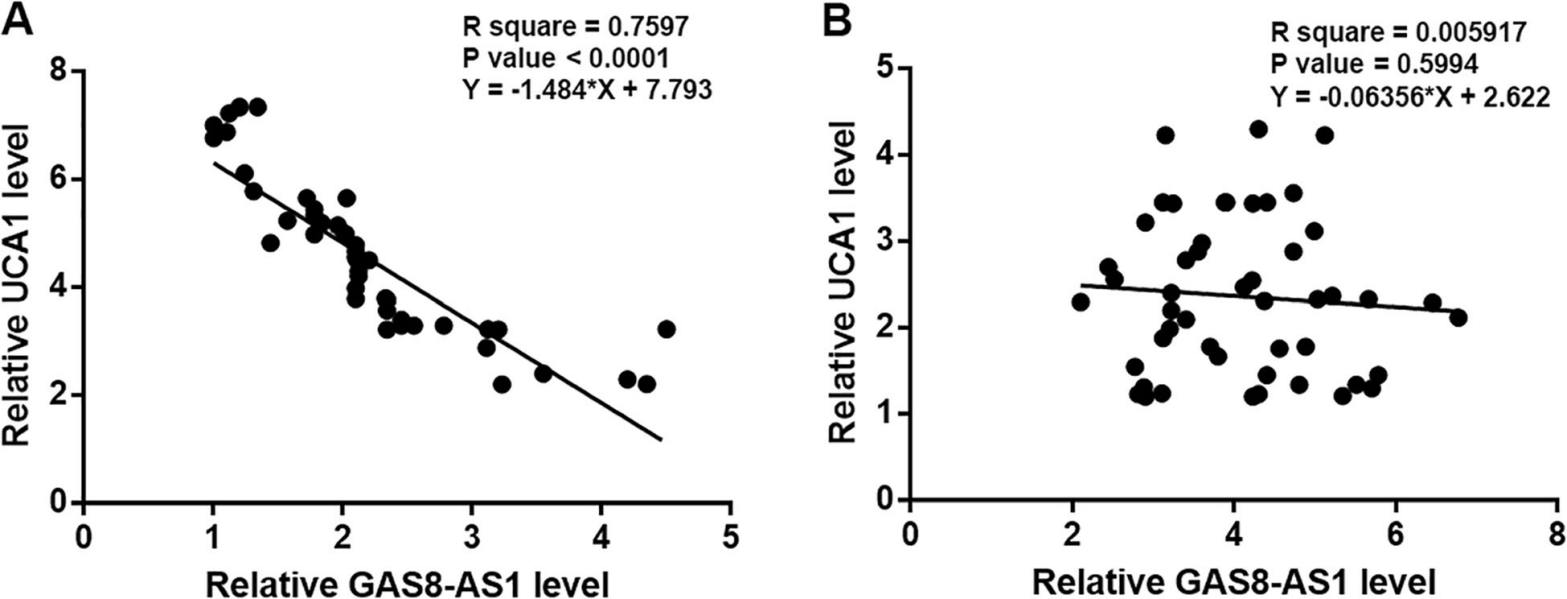
GAS8-AS1 and UCA1 were inversely correlated in OS tissues. Linear regression analysis showed that GAS8-AS1 and UCA1 were inversely correlated in OS tissues (**a**), but not in non-cancer tissues (**b**)

Correlations between the expression levels of GAS8-AS1 and UCA1 were analyzed by linear regression. The results showed that the expression levels of GAS8-AS1 and UCA1 in OS tissues were inversely and significantly correlated (Fig. 3a). In contrast, there was no significant correlation between the expression levels of GAS8-AS1 and UCA1 in non-cancer tissues (Fig. 3b).

### Overexpression of GAS8-AS1 led to downregulated UCA1

**Fig. 4:**
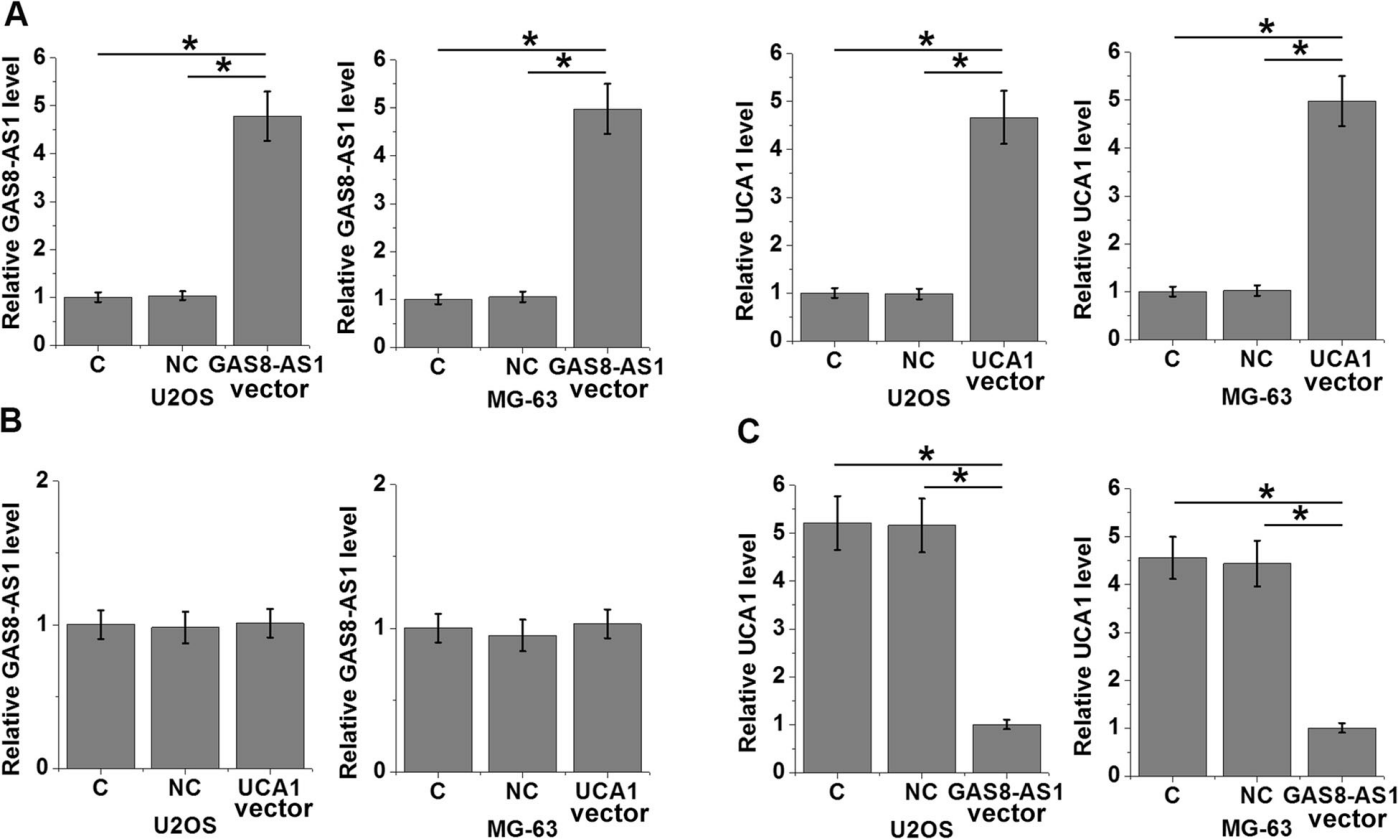
Overexpression of GAS8-AS1 led to downregulated UCA1. Compared to two controls (control, C and negative control, NC), expression levels of GAS8-AS1 and UCA1 were significantly upregulated at 24 h after the transfection of GAS8-AS1 and UCA1 expression vectors (**a**). Overexpression of UCA1 had no effect on GAS8-AS1 expression (**b**), while overexpression of GAS8-AS1 led to downregulated UCA1 in OS cells (**c**) (*p < 0.05)

GAS8-AS1 and UCA1 expression vectors were transfected into U2OS and MG-63 cells. Compared to the two control groups (C and NC), expression of GAS8-AS1 and UCA1 were significantly upregulated at 24 h after transfection (Fig. 4a, *p* < 0.05). Compared to the two control groups, overexpression of UCA1 had no effect on the expression of GAS8-AS1 (Fig. 4b), while overexpression of GAS8-AS1 led to downregulated UCA1 in OS cells (Fig. 4c, *p* < 0.05).

### GAS8-AS1 inhibited OS cell migration and invasion through UCA1

**Fig. 5:**
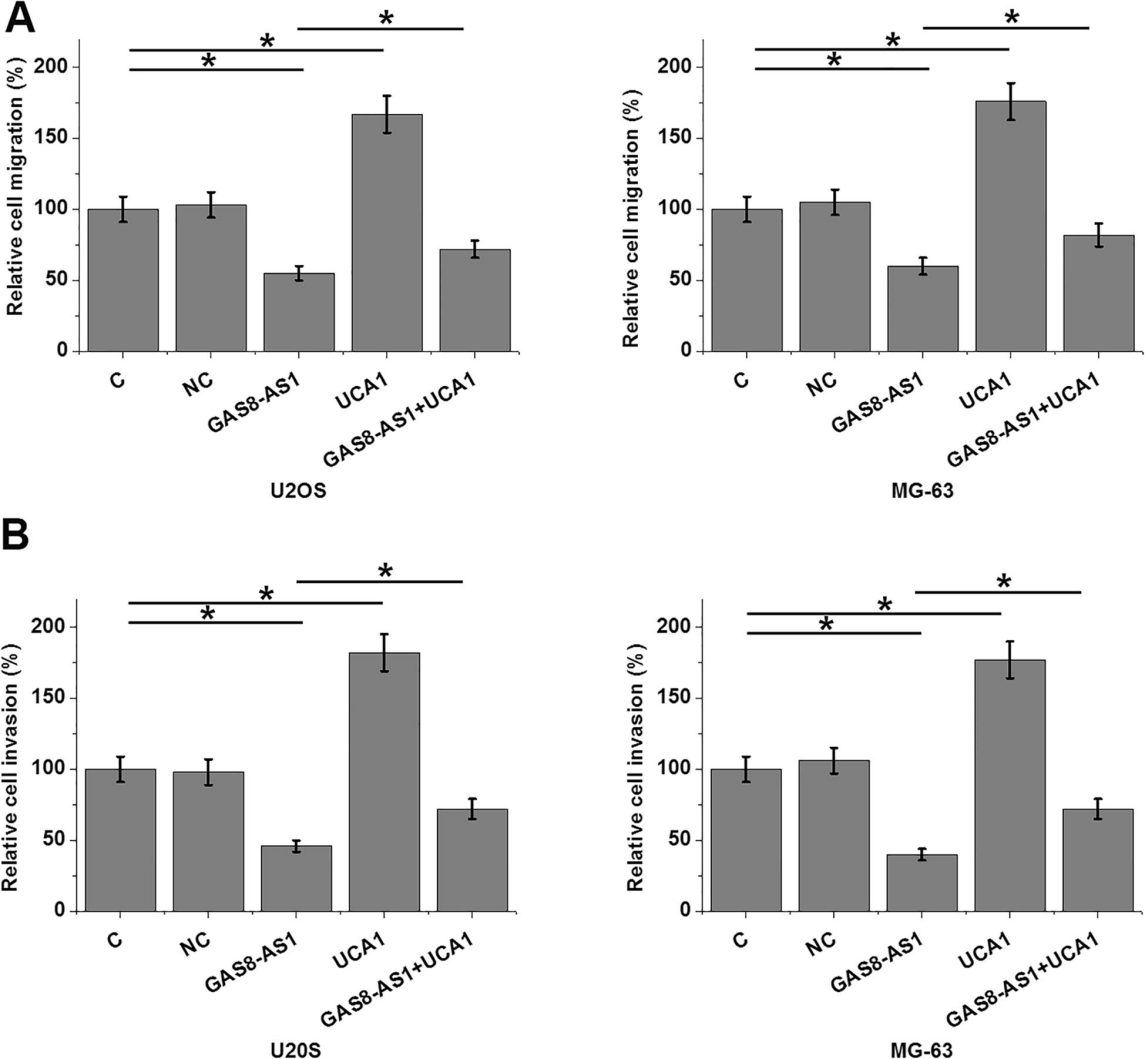
GAS8-AS1 inhibited OS cell migration and invasion through UCA1. Compared to two controls (control, C and negative control, NC), Transwell migration and invasion assay showed that overexpression of GAS8-AS1 resulted in inhibited cancer cell migration (**a**) and invasion (**b**). In contrast, overexpression of UCA1 promoted the migration and invasion of OS cells and attenuated the effects of GAS8-AS1 overexpression (*p < 0.05)

Cell proliferation assay showed that overexpression of GAS8-AS1 had no significant effects on OS cell proliferation compared to the two control groups (C and NC) (data not shown). Transwell migration and invasion assay showed that overexpression of GAS8-AS1 resulted in inhibited cancer cell migration (Fig. 5a, *p* < 0.05) and invasion (Fig. 5b, *p* < 0.05). In contrast, overexpression of UCA1 promoted the migration and invasion of OS cells and attenuated the effects of GAS8-AS1 overexpression.

## Discussion

lncRNA GAS8-AS1 has been characterized as a tumor suppressor in papillary thyroid carcinoma [11, 12]. The key finding of the present study is that GAS8-AS1 is downregulated in OS and may play a role as tumor suppressor in OS by downregulating lncRNA UCA1, which promotes OS progression.

Accurate prognosis is critical for the design of individualized treatment for cancer patients, especially for those who were diagnosed at advanced stages [15]. Up to date, many prognostic biomarkers have been developed for OS, such as ERCC polymorphisms and Cyr61 expression [16, 17]. In recent years, circulating biomarkers, such as blood circulating lncRNAs have been attracting more research attentions due to the non-invasive nature [18]. A recent study reported that plasma GAS8-AS1 was downregulated in Chinese patients with papillary thyroid carcinoma, and downregulated GAS8-AS1 provided novel diagnostic insights [12]. Our study showed that low expression level of GAS8-AS1 in OS tissue is closely correlated with the poor survival of OS patients. In view of the fact that OS is usually diagnosed by pathological biopsy, expression of GAS8-AS1 may serve as a potential prognostic marker for OS.

lncRNAs mainly function by affecting the expression of downstream genes at different levels [19]. However, the interaction between different lncRNAs has not been well studied. In the present study we found that GAS8-AS1 was an upstream inhibitor of UCA1 in OS cells, and this interaction is involved in the regulation of migration and invasion of OS cells. It is known that UCA1 can participate in cancer biology by interacting with multiple signaling pathways, such as the FGFR1/ERK signaling pathway and the TGF-β pathway [20, 21]. Therefore, there might be other signaling pathways serving as mediators between UCA1 and GAS8-AS1. Our future studies will investigate this hypothesis.

## Conclusion

In conclusion, GAS8-AS1 was downregulated in OS and GAS8-AS1 overexpression may inhibit OS by downregulating UCA1.

## Data Availability

The datasets used and/or analyzed during the current study are available from the corresponding author on reasonable request.

## Abbreviations

lncRNAs: Long non-coding RNAs
OS: osteosarcoma
